# Social work contact in a UK cohort study: Under-reporting, predictors of contact and the emotional and behavioural problems of children

**DOI:** 10.1016/j.childyouth.2020.105071

**Published:** 2020-08

**Authors:** Meng Le Zhang, Andrew Boyd, Sin Yi Cheung, Elaine Sharland, Jonathan Scourfield

**Affiliations:** a ICOSS Building, 219 Portobello, Sheffield S1 4DP, United Kingdom; b Data Linkage and Information Security Manager, Oakfield House, Bristol BS8 2BN, United Kingdom; c Cardiff University, Glamorgan Building, Cardiff CF10 3WT, United Kingdom; d Sussex University, Room 108, Essex House, Falmer, Brighton BN1 9QQ, United Kingdom; e CASCADE, School of Social Sciences, Cardiff University, 1-3 Museum Place, Cardiff CF10 3BD, United Kingdom

**Keywords:** Social work, Emotional and behavioural problems, ALSPAC

## Abstract

**Background:**

There is little evidence on the degree of underreporting of social work contact in survey studies. There are also few studies about the emotional and behavioural problems of children in families who have contact with social workers, despite the adversities they face.

**Objective:**

We examine underreporting of social work contact; the predictors of social work contact with families; and links between social work contact and emotional and behavioural outcomes for children.

**Participants and setting:**

The Avon Longitudinal Study of Parents and Children follows the health and development of 14,062 children born in 1991–2 to women living in and around the City of Bristol. The study includes self-reported information about mothers’ contact with social workers and children’s emotional and behavioural outcomes using the Strengths and Difficulties Questionnaire (SDQ).

**Methods:**

Self-reporting of social work contact in ALSPAC is compared with administrative data from the child protection register to examine rates of underreporting. We use a fixed-effects model to account for measurement error in estimating the association between social work contact and potential predictors, namely gender of the child and maternal factors: marital status, trouble with law, hospitalisation, cannabis and alcohol use, employment, financial difficulty and experience of cruelty from a partner. SDQ scores are explored using linear regression with lagged indicators of social work contact.

**Results:**

The probability of a false negative for self-report of social work contact ranged from 22% to 34% across three survey waves. Mothers who married within the last 12 months were less likely to receive social work contact (OR: 0.13, CI95%: 0.01–1.34). Those who had either been hospitalised (OR: 1.52, CI95%: 1.01 – 2.28) or increased their rate of alcohol consumption (OR: 2.14, CI95%: 0.91 – 5.07) within the last 12 months were more likely to receive such contact. Overall children whose mothers report social work contact were much more likely to have emotional and behavioural problems within the first seven years of their lives (p < 0.01).

**Conclusion:**

There is potentially a high degree of underreporting of social work contact in social surveys and cohort studies. Researchers should adopt methods to account for this issue in the future. The risk of emotional and behavioural problems is greater among children whose mothers have had contact with social workers compared to other children with seemingly similar adversities.

## Highlights

• Examines children whose mothers had social worker contact in an English cohort study.
• Found substantial levels of underreporting of social worker contact in the study.
• Identified factors associated with an increased chance of social worker contact.
• Worse child emotional and behavioural problems were associated with contact.

## 1 Introduction

Many studies have focussed on the relationship between emotional and behavioural difficulties in children and a range of factors such as family poverty and single parenthood (Bergeron et al., 2000, Sabates and Dex, 2015). These studies have helped identify potentially vulnerable or disadvantaged groups of children in the general population. However, there is little literature looking at mental health outcomes for children whose parents have been in contact with a social worker, although these are likely to be children experiencing adversities. It is known that children in foster care or residential homes have higher rates of psychiatric disorders compared to the general population (Ford, Vostanis, Meltzer, & Goodman, 2007). These cases represent the most vulnerable group of children who come into contact with social workers, usually as a result of child abuse or neglect. However, the generic profession of social work also deals with individuals and families made vulnerable by a range of factors: mental health issues, alcohol, drug or other substance abuse, domestic abuse, social exclusion, learning difficulties and so forth. Children in families facing these issues may not be at immediate severe risk of significant harm warranting a child protection plan or placement in out-of-home care, but may be especially vulnerable and defined as ‘in need’. Recent estimates from the Office of the Children’s Commissioner for England (2019), for example, indicate that whilst 73,500 children in England were in out-of-home care at the end of 2017/18, and a further 52,640 subject to child protection plans, together they constituted less than one-third of the total 397,430 children designated ‘in need’ and eligible to receive social work support.

The primary objective of this paper is to examine the various events and circumstances that precede mothers’ contact with social workers, and the association between that contact and children’s mental health outcomes. This is done with caution in recognition of the challenges firstly of capturing and measuring child and family adversities, vulnerabilities, assets, resilience or social care outcomes (Children’s Commissioner for England, 2018a, Children’s Commissioner for England, 2018b, Forrester, 2017) and secondly, their complex and compounding intersections (Davidson, Bunting, & Webb, 2012). Thirdly, we are alert to the ethical and political, as well as practical, pitfalls of risk calculations, prediction and over-interpretation (France et al., 2010, White et al., 2019). There is nonetheless value, with due caution, in drawing on existing longitudinal data to see what can be learned about social work intervention and its outcomes for vulnerable children and families. We use the terms ‘predictors’ and ‘outcomes’ throughout this paper without any necessary or direct attributions of causality.

## 2 Background

Social workers are central figures among the frontline professionals who support and protect vulnerable individuals and families. Their involvement may be sought voluntarily or received involuntarily if there is a potential risk of significant harm. Social workers commonly also work alongside other professionals, such as health and education staff, to promote service users’ welfare and ensure their safety. However relatively little is known either about the adversities facing users of routine social work services or about their outcomes compared to the general population, since there are few large-scale studies on this topic, especially in the United Kingdom. There is very limited coverage of social work in UK cohort and panel studies (Maxwell, Scourfield, Gould, & Huxley, 2012) and quantitative research capacity among UK social work academics is under-developed (Sheppard, 2015). The UK does not have the data infrastructure equivalent to countries which have either a dedicated nationally representative study of social work service users, such as the National Survey of Child and Adolescent Well-being in the US (Bellamy, 2009), or a register dataset that includes information on child welfare contact, as in the case of Sweden (Vinnerljung et al., 2006, Vinnerljung et al., 2006). Where information is available and analysed, there is evidence to suggest that outcomes for those in contact with social workers, or their children, are generally worse than their general population counterparts. Though we must be cautious about inferring causality, not least due to limitations of observational data within each dataset, these findings hold once known adversities are taken into account. Teenagers who have had social worker contact due to problem behaviour are less likely to achieve benchmark standards of educational attainment (Henderson, Scourfield, Cheung, Sharland & Sloan, 2016). Parents who use social work services have worse mental health outcomes over time and report poorer wellbeing for their children (Henderson, Cheung, Sharland, & Scourfield, 2015). European studies show that young people who have been in contact with social workers are more at risk of suicide attempts, committing criminal offences and having psychiatric disorders (Vinnerljung et al., 2006, Vinnerljung et al., 2006).

Since social work services target vulnerable individuals and families and given the known (albeit often mediated) relationship between adversities and children’s mental health outcomes (Bergeron et al., 2000, Arseneault et al., 2011, Sabates and Dex, 2015), it would not be surprising to find that these children faced worse outcomes than others in the general population. Social work service users live in very deprived circumstances (Sidebotham et al., 2002, Bywaters et al., 2020) and the link between deprivation and poor health is well known (Marmot, 2005). However, there are also reasons to believe that the degree of risk facing these children is more than just the sum of the adversities they encounter. Multiple adversities – at social as well as individual and family levels - may have complex interactions with each other that compromise assets and resilience and compound any negative effects on children’s wellbeing and development (Evans, Li, Whipple, & Sepanski Whipple, 2013). Children whose families who have had social work contact may be particularly at risk of developing, or already having, emotional and behavioural health problems even when compared to other groups of vulnerable children.

Given a relative lack of detailed administrative data infrastructure to identify social work service users in the UK, researchers must rely on data collected from other sources such as longitudinal studies. Examples include the Millennium Cohort Study (MCS) (Zhang, Henderson, Cheung, Scourfield, & Sharland, 2016), the Longitudinal Survey of Young People in England (Henderson, Scourfield, Cheung, & Sharland, 2016) and—the focus of this paper— Avon Longitudinal Study of Parents and Children (ALSPAC) (Sidebotham et al., 2002). Even if high-quality administrative data were to exist on social work service users, its purposes would be not aimed towards research and thus would lack many variables of interest to researchers. For instance, in other fields such as public health, administrative information on NHS service users is available in the UK but the range of common socio-demographic variables (such as social class or education) are missing. We acknowledge there is currently considerable effort to create information-rich datasets using data linkage but—for the foreseeable future—self-reported data on service usage will remain the norm. We suspect this will be true in many other countries and therein lies an additional problem of misreporting of social work contact in survey studies.

Social work contact may be misreported in surveys due to several reasons. First, there is could be some degree of random recall error—especially if participants are asked to recall over longer periods. Second, misattribution bias could be a factor as people may not recognise social workers as such or have brief contact with social workers through other family members who are service users. Third, on this socially sensitive topic, parents may under-report due to social desirability bias. Consequently, the rate of underreporting may be higher for those who receive social work contact for more severe reasons such as child maltreatment.

### 2.1 The current paper

The misreporting of social work contact can confound the ability of any analysis where the aim is to determine either (i) predictors associated with social work contact or (ii) outcomes associated with social work contact. This paper considers both predictors and outcomes. In this paper (and other similar studies), we are careful to not claim causality –especially given our reliance on observational data. We are nonetheless interested in whether certain factors or life events remain predictors of social work contact or behavioural outcomes even in the presence (or absence) of other factors. This includes factors that are both observed and unobserved by researchers. Since predictors may still indicate particular at-risk or vulnerable populations, it is desirable for the statistical relationship between predictors and social work contact (or contact and outcomes) to be unmediated by data quality issues.

We make use of the Avon Longitudinal Study of Parents and Children (ALSPAC), a UK birth cohort study. Information from the statutory child protection register (CPR) has been linked to ALSPAC data and results from an analysis of these linked data presented previously (Sidebotham and Golding, 2001, Sidebotham et al., 2002, Sidebotham and Heron, 2003, Sidebotham and Heron, 2006). In this paper, we exploit use this linkage to empirically explore the degree of false negatives in the reporting of social work contact amongst families with a record on the CPR. We discuss the potential issues with that underreporting can cause for statistical analyses and how longitudinal data analysis using fixed effects can alleviate issues related to misreporting. Finally, in the absence of a previous analysis on this topic using ALSPAC, we analyse the association between social work contact and emotional and behavioural health. For our final analysis, we recognise the limits of such an analysis given issues of misreporting.

## 3 Materials and methods

### 3.1 Overview

As mentioned earlier, there are many reasons for the mismeasurement of social work contact (henceforth referred to as ‘contact’). These errors may be random or non-random and systematic. Examples of the former are recall errors due to random events on the day of data collection. Non-random error can occur if more severe cases—such as contact related to child maltreatment—are under-reported. Another example of non-random error occurs if some unobserved traits are correlated with both underreporting and our observed predictors of contact. When contact is the outcome of interest, random measurement error only leads to inefficiencies in estimation whilst non-random errors lead to bias in the real association between predictors and contact. Essentially non-random errors can be treated as confounders which can be accounted for in special cases given access to longitudinal data.

Intuitively, we can describe the research design like this. If the propensity to underreport is driven by unobserved factors—such social desirability—linked to a person that does not vary over time then we can use the same person as their own ‘controls’ to ask (paraphrasing Allison & Christakis, 2006, 159): Given that contact occurred during this period, why did it not occur the period before? Did something occurring during this period that did not occur in others? If factors related to underreporting have not changed, these factors cannot be responsible.

This is the fundamental principle behind fixed-effects methods and we show how a particular variant—the fixed-effect logit model—can be used to predict contact. Unfortunately, when contact itself is a predictor of other outcomes—such as behaviour—the same family of methods cannot be reliably used to alleviate measurement error.

After explaining the problem of measurement error, we then use linkages between ALSPAC and the CPR to identify rates of underreporting amongst mothers investigated for child maltreatment when their child was aged 21 months, 33 months and 73 months respectively. Then we use the fixed-effect logit model to predict whether changes in risk factors are associated with changes in contact in with mothers of children aged between 21 and 33 months. Our research design uses changes in the lives of children and mothers to predict social work contact. These changes can correspond to life-changing events—such as marriages or separations. Finally, we conduct an exploratory analysis of children at age 42 and 81 months comparing those mothers have not had contact in a previous period.

### 3.2 The problem of measurement error

First, we will introduce the problem of measurement error in terms of a numeric outcome $Y^{*}$ in analyses of only two time periods. For individual i during the period t (where t=1or2), the outcome $Y_{\mathrm{it}}^{*}$ is a function of:

$$Y_{\mathrm{it}}^{*}=\beta_{0t}+\beta_{1}X_{\mathrm{it}}+u_{i}+e_{\mathrm{it}}$$

$X_{\mathrm{it}}$ is a predictor which can vary over time. There also exist unobserved characteristics or adversities $u_{i}$ which do not vary over time and may or may not be correlated with $X_{\mathrm{it}}$. $e_{\mathrm{it}}$ is the random error term which may randomly vary in the outcome not correlated with the other terms. The Greek letters are parameters to be estimated. $\beta_{0t}$ is the intercept term whilst out actual parameter of interest is $\beta_{1}$ which tells us the relationship between our predictor X and outcome $Y^{*}$. In particular, we want to know direction and size of $\beta_{1}$. In a model with more than one predictor (i.e. X and Z), we may at least desire to know the relative size of these parameters (i.e. is $\beta_{1}>\beta_{2}$).

We wish to measure $\beta_{1}$ or at least its properties however measurement error occurs when we do not observe $Y^{*}$. Instead, we have an imperfect measure Y and measurement error is defined as the difference between Y and $Y^{*}$:

$$Y_{\mathrm{it}}^{*}-Y_{\mathrm{it}}=w_{i}+v_{\mathrm{it}}$$

The measurement error is split into two components: $w_{i}$ is the measurement error in the reporting of $Y^{*}$ that remains consistent for each person i over time and $v_{\mathrm{it}}$ is random measurement error that can vary over time. The relationship between Y and X (and other variables) is:

$$Y_{\mathrm{it}}=\beta_{0t}+\beta_{1}X_{\mathrm{it}}-w_{i}-v_{\mathrm{it}}+u_{i}+e_{\mathrm{it}}$$

A regression of Y on X will yield biased estimates of $\beta_{1}$ if X is correlated with either the $w_{i}$ or $u_{i}$. In short, $w_{i}$ and $u_{i}$ are unobserved omitted variables from the regression equation (i.e. confounders).

### 3.3 Fixed-effect models and measurement error

If we assume, as in the above example, that the confounders do not change over time then we can use fixed-effect models to account for bias. The average of Y across all periods for individual i is equal to:

$$\bar{Y_{i}}=\bar{\beta_{0}}+\beta_{1}\left({\bar{X}}_{i}\right)-{\bar{w}}_{i}-{\bar{v}}_{i}+{\bar{u}}_{i}+{\bar{e}}_{i}$$

where the bar notation denotes averaging over all periods. The average of the time-varying errors u and e will be zero. Since the time-invariant confounders do not change (i.e. $u_{i1}$ = $u_{i2})$then the average ${\bar{u}}_{i}$ is simply equal to $u_{i}$. This results in:

$$\bar{Y_{i}}=\bar{\beta_{0}}+\beta_{1}\left({\bar{X}}_{i}\right)-w_{i}-v_{i}$$

If we take the difference between $Y_{\mathrm{it}}$ and ${\bar{Y}}_{i}$ (or demean Y):

$$Y_{\mathrm{it}}-{\bar{Y}}_{i}=\left(\beta_{0t}-{\bar{\beta }}_{0}\right)+\beta_{1}\left(X_{\mathrm{it}}-{\bar{X}}_{i1}\right)-\left(w_{i}-w_{i}\right)+\left(u_{i}-u_{i}\right)-v_{\mathrm{it}}+e_{\mathrm{it}}$$

$$=\left(\beta_{0t}-{\bar{\beta }}_{0}\right)+\beta_{1}\left(X_{\mathrm{it}}-{\bar{X}}_{i1}\right)-v_{\mathrm{it}}+e_{\mathrm{it}}$$

We are left with an estimating equation without the time-invariant confounders $w_{i}$ and $u_{i}$. We can estimate all the parameters from this model using a regression of the demeaned value of Y on the demeaned values of X $\left(i.e.\;X_{\mathrm{it}}-{\overset{¯}{X}}_{i1}\right)$ although steps have to be taken to adjust the standard error. Whilst the FE estimator is usually used to solve the problem of unobserved confounding risk factors ($u_{i}$) it can also be used to address measurement error (Wooldridge, 2002, 321).

The basic logic of the fixed effect / first difference estimator can be extended to cases where $Y^{*}$ is a binary outcome using a fixed-effect logit estimator (otherwise known as a conditional logit model)(for a full explanation see Wooldridge, 2002, Chamberlain, 1980). There are several differences between the fixed-effect linear and logit models. For our purposes, it is worth noting that the FE logit model uses far fewer cases than the linear FE model. The linear FE estimator for two time periods uses information all cases present at both periods. On the other hand, the FE logit uses information from cases where there is a change in the recorded outcome between two time periods (e.g. one period there is contact and another period where there is not).

#### 3.3.1 Limitation of fixed-effect methods

The downside of fixed effects (FE) methods is a decrease in statistical efficiency (compared to using pooled cross-sectional data). This limitation is further exacerbated for the fixed-effect logit model when discards cases where there is no change in outcomes. Another downside of fixed-effect models is that we cannot estimate the effects of predictors that do not vary over time. For instance, if Z was a predictor that did not change over time (e.g. sex of the child) such that:

$$Y_{\mathrm{it}}=\beta_{0t}+\beta_{1}X_{\mathrm{it}}+\beta_{2}Z_{i}-w_{i}+u_{i}-v_{\mathrm{it}}+e_{\mathrm{it}}$$

We can see that $Z_{i}$ drops out of the model along with $w_{i}$ and $u_{i}$ once we start differencing (i.e. $Z_{i}={\bar{Z}}_{i}$). However, it is still possible to estimate the interaction between Z and period T where T is equal to 0 at time t=1 and 1 if t=2. This interaction may indicate the existence of difference in time trends—for instance, if mothers of male children are more likely to receive social work contact as their child grows.

In logistic regression, average marginal effects are often used as another metric of effect size instead of the more abstract odds ratio. Another limitation of the FE logit model is that average marginal effects cannot be calculated since the fixed effects (e.g. u and w) are not estimated. For our analysis, we compare the relative size of estimated odds ratios—within the same statistical model—as a measure of the predictive effect size for each predictor.

Finally, the advantages of FE becomes less clear when contact is used as a predictor of other outcomes. In this case, any measurement error in contact—even if it is random—will induce bias in cross-sectional data. This is may be exacerbated further in FE methods depending largely on the relative size of the time-varying and time-invariant components of measurement error ($u_{i}$ and $v_{\mathrm{it}}$). Generally, it is believed that differencing or FE in these cases increase bias (Wooldridge, 2002, 608–9).

In short, FE methods can help when there is measurement error in the dependent variable albeit at the expense of some limitations and statistical efficiency (Woolridge, 2002). Given the extent of potential misreporting, we choose to apply FE methods to estimate predictors of contact. However, given the issue of measurement error in predictors, we only use a standard linear model with (lagged) indicators of contact to predict emotional and behavioural health.

### 3.4 Data

We make use of the Avon Longitudinal Study of Parents and Children (ALSPAC), a UK birth cohort study, to examine the characteristics of mothers and the health outcomes for children. ALSPAC recruited pregnant women resident in and around the City of Bristol, UK, with expected dates of delivery from 1st April 1991 to 31st December 1992. A total of 14,541 expectant mothers initially enrolled in ALSPAC and had either returned at least one questionnaire or attended an ALSPAC “Children in Focus” clinic by 19th July 1999. Of these initial pregnancies, there were a total of 14,062 live births and 13,988 children who were alive at 1 year of age (Boyd et al., 2013). Ethical approval for the study was obtained from the ALSPAC Ethics and Law Committee and the Local Research Ethics Committees. Timelines of the data collection and ALSPAC response rates are given in Boyd et al. (2013).

#### 3.4.1 Linkage to child protection register

This allows us to know whether a child was investigated for possible (including pre-natal) abuse or neglect and whether they were subsequently placed on the child protection register between 1 January 1991 and 31 December 1998. In these more serious situations, the records serve as a reliable indicator of social work contact, since social workers are always involved in these investigations, and their involvement will certainly have been known to the mother. The timing of any investigations and registrations is known, but detailed case-specific information has not been extracted for this study. Information linked to the child protection register is available up until the child is aged 6 for cases that were subsequently lost to follow-up, but not for those who formally withdrew from the study. However, the practical application of this information for longitudinal research is limited, since the overwhelming majority of families where there were child protection concerns were eventually lost to follow-up.

ALSPAC do not permit the sharing of these sensitive records beyond the small ALSPAC Data Linkage Team (which includes author Boyd) who operated using secure Data Safe Haven principles. To facilitate this investigation Boyd created a synthetic version of the records (using the synthpop package in R, Nowok, Raab, Snoke, & Dibben, 2016). Author Zhang developed analytical code using synthetic data to extract relevant statistics related to misreporting. In turn, the analytical code was run using the real data within the ALSPAC secure environment by Boyd and forms the basis for our final results. Finally, aggregate outcomes were checked for disclosure risk before inclusion in this paper.

#### 3.4.2 Self-reported contact with social workers

When the study child was aged 21 months, mothers were asked: “In the past year please indicate whether you have had contact with any of the following, for whatever reason?” The answer categories cover several different professions including social worker. The same question was repeated when the child was 33 and 73 months old although at 33 months mothers were asked about contact during the preceding 18 instead of 12 months. An additional question about contact with social services (as opposed to social workers) was also asked at age 73 months. ALSPAC does not contain any other information about the type of contact that mothers had, its duration, intensity or quality. Nor does it indicate whether the contact was voluntary or involuntary, its reasons or intended purpose. Some reported social work contact may have been due to the social needs of adult family members, such as a frail older person.

#### 3.4.3 Predictors of contact and other outcomes

Previous research has helped to identify adversities and vulnerabilities that may affect the likelihood of mothers receiving social worker contact and may impact on children’s mental and behavioural outcomes (Davidson et al., 2012, Sabates and Dex, 2015, Sidebotham and Golding, 2001, Mulder et al., 2018). For predicting contact, our selection of variables includes events that change over time rather than static factors favoured by previous research on predicting child maltreatment by Sidebotham and Heron (2006). This means that time-invariant characteristics — such as sociodemographic background — were not included in the analysis. The only exception to this is the gender of the child which was included to reflect potentially different time trends in the probability of having contact. Previous research (Zhang et al., 2016) suggests that contact is more likely if the young person is male, once other circumstances are taken into account.

The exclusion of time-invariant predictors is due to the limitation of fixed-effect models which cannot be used to estimate the effects of time-invariant effects on an outcome. Instead, this paper focuses on dynamic events that are associated with social work contact which are also less emphasised in the previous series of papers by Sidebotham and collaborators. To maintain consistency in our analysis, we also use the same set of predictors when exploring the relationship between maternal social worker contact and emotional and behavioural outcomes. This set of variables has large overlaps with a previous analysis exploring the relationship between social worker support and SDQ in the Millennium Cohort Study (Zhang et al., 2016, 775).

Our predictive model of contact includes risk factors that are consistently reported at two ALSPAC waves. These include dynamic factors include changes in the reporting of any emotional cruelty to the mother or child; whether the mother has been made homeless; whether she has experienced depression; her marital or partnership status; whether she has been hospitalised in the previous 12 or 18 months; her employment status; whether she ‘was in trouble with the law’; and any financial difficulties in the household. Changes in risk-taking behaviour, such as whether the mother drinks every day or takes cannabis, are also included. The wording of some indicators changes over time. For example, later ALSPAC questionnaires ask whether a mother has married over a period of time instead of marital status (see Table 1). The fully searchable ALSPAC study data dictionary contains details of all these adversity indicators (http://www.bris.ac.uk/alspac/researchers/data-access/data-dictionary/).

**Table 1:** Main ALSPAC sample used for children SDQ models.

Variable		Percentages	
ALSPAC variable name [% valid response]		Time 1	Time 2
**Mother Variables (Reporting periods: T1 = 33 months [Total N = 9365] | T2 = 73 months [Total N = 8531])**			
Mother has contact with social workers	No	95.8	95.9
h934 (100%) | l8004 (97%)	Yes	4.2	4.1
Mother has been depressed	No	76.5	78.1
h013 (100%) | l3011 (99.3%)	Yes	23.5	21.9
Mother's marital status	Single	10.7	
h386 (98.3%) | NA	Married more than once	8.6	
	First marriage	73.8	
	Separated/ Divorced	6.9	
Partner status	No partner	5.4	5.7
h480 (99.7%) | l6000 (99.4%)	Has partner	94.6	94.3
Mother has been in trouble with the law	No	99.4	99.6
h217 (99.2%) | l4007 (99.5%)	Yes	0.6	0.4
Mother has been hospitalised	No	75.1	86.3
h060 (99.6%) | l3180 (99.4%)	Yes	24.9	13.7
Mother has taken cannabis	No	96	95.8
h037 (99.1%) | l3042 (99.4%)	Yes	4	4.2
Mother is in paid employment	No	56	
H667 (97.7%) | NA	Yes	44	
Mother drinks at least once per day	No	89.3	99
h723 (99.2%) | l3022 (99.5%)	Yes	10.7	1
Mother's age birth of child	20 and over	97.9	98.2
	Under 20	2.1	1.8
Mother's has been married	No		99
NA | l4025 (99.2%)	Yes		1
Mother's has been divorced	No		97.8
NA | l4008 (99.4%)	Yes		2.2
Mother has got a new job	No		78.6
NA | l4031 (98.9%)	Yes		21.4
Mother has lost a job	No		96.9
NA | l4014 (99.2%)	Yes		3.1
Mother has been in major financial difficulty	No		90.6
NA |l4024 (99.2%)	Yes		9.4

**Child’s Variables**			
Gender of child	Female	48.6	48.8
kz021 (100%)	Male	51.4	51.2
Child’s SDQ score [T1 = 47 months | T2 = 81 months]	Mean	8.82	7.26
j555f (94.7%) | kq345f (85.7%)	Standard deviation	4.56	4.69

**Other Variables**			
Partner has been emotionally cruel to mother or child (derived) (T1 = 33 months | T2 = 73 months)	No	88.9	92.4
h246 (99.1%) + h247 (99.1%) | l4036 (99%) + l4037 (99.1%)	Yes	11.1	7.6
Financial difficulty score (73 months only)	Mean	2.99	
h735 (99.3%)	Standard deviation	3.59	
Total N (after case-wise deletion)		7951	6304

#### 3.4.4 Self-reported measure of a child’s emotional and behavioural problems

The measure of the child’s mental and emotional health used in ALSPAC is the standardised Strengths and Difficulties Questionnaire (SDQ, Goodman, 1997), completed by the primary parent or caretaker (almost always the mother) when the child is aged 42 and 81 months. The SDQ has been shown to have high specificity and modest sensitivity for detecting psychiatric disorders (Goodman, Ford, Simmons, Gatward, & Meltzer, 2000). Its target population is children aged 4–16, but an age-appropriate version is also available for children aged 3. The questionnaire contains five discrete subscales that capture the prevalence of children’s emotional symptoms, conduct problems, hyperactivity, peer relationship problems and prosocial behaviour. Scores (ranging from 0 to 10) for each subscale are given by responses to five separate questionnaire items. We use the sum of subscale scores (excluding prosocial behaviour) as a general indicator of mental and emotional health; higher scores are associated with a greater prevalence of emotional and behavioural problems (Kelly et al., 2009).

## 4 Statistical analysis

### 4.1 Predicting contact with social workers

In a previous study, Sidebotham et al. (2001) used ALSPAC to examine whether certain adversities or risk factors predict the prevalence of child protection investigation and registration. For our purposes, there are two limitations to this. First, there is no indication of temporal order in the Sidebotham et al. analysis—predictors could be events that had occurred after an investigation. Second, we are seeking to identify predictors for the full range of routine social work contact with vulnerable individuals and families—not just the proportion who are at the more severe end involving an investigation of child maltreatment or a child protection plan.

We use the information on social work contact when the child is 21 and 33 months of age. These two periods are relatively close in time and this strengthens our assumptions that any unobserved measurement error would remain invariant. At 21 months the mother is asked if she has had social work contact in the past 12 months, whilst at 33 months the reporting period is in the past 18 months. There are clear overlaps in the reporting; mothers who reported social work contact in the past 18 months when the child is aged 33 months could be referring to contact previously reported when the child was aged 21 months. This results in some cases being not included in the analysis if the child is aged 33 months and contact had occurred not occurred in the last 12 months BUT had occurred in the last 18 months. We envision that this is a small minority of cases and that the cause of this omission is due to overlaps in reporting rather than the circumstance of the family (i.e. uncorrelated with our predictors). This is unlikely to bias the results of the fixed-effects model but it does cause a loss of statistical power. By looking at general social work contact, we are extending the scope of previous studies by including families with children who may be vulnerable but not necessarily at as significant risk as those on the child protection register.

### 4.2 Exploratory analysis of children’s outcomes

We aim to estimate the predicted difference in emotional and behavioural problems between children whose mothers had social work contact and those whose mothers did not—after accounting for observed adversities known to be associated with poorer outcomes (Sabates & Dex, 2015). To do this we regress total SDQ score reported at 42 and 81 months using linear regression onto the list of adversities described earlier, and on to the indicator of whether the child’s mother had contact with a social worker in the previous ALSPAC wave. For the 42-month model, explanatory variables were taken from questionnaire responses at 33 months and, where appropriate, covered events that had happened in the last 12 months. For the 81-month model, the explanatory variables covered events that occurred since the child was five years of age (apart from social worker’s contact which covers the last 12 months). The samples and predictors used for the two SDQ models are described in Table 1.

Throughout our analysis, cases with missing data were excluded. In the full ALSPAC sample, 10,063 mothers responded to the 42-month questionnaire whilst 8515 did so to the 81-month questionnaire. After accounting for missing data we are left with 7951 cases at 42 months and 6304 cases at 81 months. The levels of social work contact in our samples are roughly similar to that found in the complete ALSPAC sample (~4%). As a sensitivity check, we used multiple imputation using the Amelia package in R for cases of item non-response (Honaker, King, & Blackwell, 2009). We did not find any substantively different results using multiple imputation. This is likely due to the fact that ALSPAC has very high levels of item response and missingness is largely due to unit non-response (i.e. sample attrition; see Table 1).

As discussed, children whose mothers have had contact with social workers will vary in terms of vulnerability. At one end of the spectrum, for example, contact may have resulted from child maltreatment; at the other, mothers may have sought advice from social workers for reasons unrelated to their children’s well-being. Children in the latter group may not have much higher SDQ scores than other children, after accounting for multiple adversities. To investigate the possibility that mean child SDQ scores for mothers with contact are higher due to a minority of extreme cases (i.e. social worker contact for child abuse), we examine the children’s scores in the lower and upper quartiles using quantile regression. If extreme cases are driving our results then we would expect to see little or no difference in SDQ scores, after accounting for other factors, for children in the lowest quartile. We do not explore SDQ scores beyond these timeframes due to sample attrition and the lack of reporting for social work contact in later ALSPAC waves.

## 5 Results

### 5.1 Reporting of social work contact

As noted earlier, self-reported measures of social work contact may well underestimate the actual level of contact. This has implications for the interpretation of findings from this study and others (e.g. Zhang et al., 2016). To estimate the extent of reporting bias, we calculated the proportion of mothers whose family had a child protection register record during relevant periods and who also self-reported social worker or social services contact. We compared the child protection register data with ALSPAC questionnaire responses over three periods (in the 12 months before the 21-month questionnaire, 18 months before the 33-month questionnaire, and 12 months before the 73-month questionnaire).

We linked 13,253 pregnancy units to the child protection register dataset originally extracted by Sidebotham et al in 1998. Of these, there were 193 linked investigations and 81 subsequent investigations. In total, 221 unique pregnancy units had a record on the child protection register indicating contact with a social worker on at least one occasion (1.7% of total units). These numbers differ from those reported in Sidebotham and Golding, 2001, Sidebotham et al., 2002, Sidebotham and Heron (2003) and Sidebotham and Heron (2006) whose papers were written while the reported ALSPAC denominator was in flux, and the authors’ own reported denominator changed across the four papers (Sidebotham et al., 2002; Sidebotham and Heron, 2006). Our reported denominator also excludes participants who have formally withdrawn from the study and those who have dissented to ALSPAC’s use of their Health and Social Care records. In this assessment, 9234 participants responded to the 21-month questionnaire and were included in the child protection register linkage denominator; 8795 33-month respondents were included, and 7680 73-month respondents were included. Of these, the probability of a mother with record linked to the CPR reporting a false negative response concerning social worker contact in the preceding 12 months (or 18 months for the 33-month questionnaire) was 22% in the 21-month questionnaire; 34% in the 33-month questionnaire, and 22% in the 73-month questionnaire. Precise percentages have been suppressed for disclosure control reasons.

### 5.2 Predictors of contact with social workers

Since the FE logit relies on cases where social work contact was established at one time period and not another, we are restricted to relatively few cases (N = 390). In Table 2, we can see that mothers who have been hospitalised at some point in the preceding 12 months are more likely to have had contact with social workers (OR: 1.52, CI95%: 1.01–2.28). Mothers who had married for the first time within the preceding 12 months were less likely to have social work contact (OR: 0.13, CI95%: 0.01–1.34). Conversely, mothers who drink alcohol at least once per day were more likely to have had contact with social workers (OR: 2.14, CI95%: 0.91–5.07). These results are similar to our findings about the predictors of social work contact using a different longitudinal data set (Zhang et al., 2016).

**Table 2:** Fixed Effects Models of the odds (and CI95%) of social work contact.

	*Dependent variable:*	
	Raw estimate	Odds ratios
	(1)	(2)
Child is male	0.29	1.34
	(−0.13, 0.71)	(0.88, 2.03)
Mother has been depressed	0.03	1.03
	(−0.40, 0.45)	(0.67, 1.57)
Mother married for first time [Base = single and no partner]	−2.06*	0.13*
	(−4.41, 0.29)	(0.01, 1.34)
Mother married second time or later	0.55	1.72
	(−1.84, 2.93)	(0.16, 18.77)
Mother separated/ divorced	0.57	1.78
	(−0.87, 2.02)	(0.42, 7.52)
Mother unmarried with a partner	−0.23	0.80
	(−1.00, 0.55)	(0.37, 1.73)
Mother has been in trouble with the law	−0.16	0.85
	(−1.55, 1.23)	(0.21, 3.41)
Mother has been hospitalised	0.42^**^	1.52^**^
	(0.01, 0.82)	(1.01, 2.28)
Mother has taken cannabis	0.62	1.87
	(−0.57, 1.81)	(0.57, 6.14)
Mother is in paid employment	0.03	1.03
	(−0.36, 0.42)	(0.70, 1.53)
Mother drinks at least once per day	0.76*	2.14*
	(−0.10, 1.62)	(0.91, 5.07)
Mother has had financial difficulties	0.01	1.01
	(−0.06, 0.07)	(0.94, 1.08)
Partner has been emotionally cruel to mother or child	0.40	1.49
	(−0.13, 0.93)	(0.88, 2.53)
Constant	−0.15	0.86
	(−0.46, 0.16)	(0.63, 1.17)
Observations	390	390
Log Likelihood	−255.61	−255.61
Akaike Inf. Crit.	539.22	539.22
*Note:*	*p < 0.1; ^**^p < 0.05; ^***^p < 0.01	

### 5.3 Outcomes for children whose mothers had contact with social workers

Looking at children’s emotional and mental health outcomes at 42 and 81 months, descriptive statistics show that on average children whose mothers had social work contact in the preceding months had SDQ scores 2.37 (s.d. = 0.46) and 2.97 (s.d. = 0.47) higher than those without contact. These elevated scores might be expected, given that these children and their families may face multiple adversities.

However, after accounting for several circumstances and adversities, we find that those children whose mothers had social work contact are still much worse off. The estimated difference is 1.61 at 42 months and 2.24 at 81 months (Table 3, Table 4). The results show that experiences such as emotional cruelty, teenage pregnancy with study child, mothers’ depression, job loss and financial difficulties are also associated with higher SDQ scores although the effect sizes are smaller than those associated with maternal social work contact. For instance, partner cruelty to the mother or the child is associated with SDQ scores that are 0.69 higher at 42 months and 0.73 higher at 81 months. This highlights the extent to which children whose mothers have had contact with social workers for whatever reason are particularly vulnerable compared to groups who face other adversities—even after we take into account other factors. The results from the quantile regression (Table 5) show that these differences persist at different SDQ quartiles.

**Table 3:** SDQ scores at 42 months (OLS).

	Estimate	CI95%	*p*-value
(Intercept)	7.49	(6.94–8.04)	0.00
Mother has contact with social workers	1.61	(1.12–2.10)	0.00
Child is male	0.92	(0.72–1.12)	0.00
Mother has been depressed	1.35	(1.11–1.59)	0.00
Mother's marital status (ref = Never married)			
First marriage	−0.94	(−1.41–−0.47)	0.00
Second or later marriage	−0.69	(−1.04–−0.34)	0.00
Separated/ Divorced	−0.3	(−0.79–0.19)	0.23
Mother has partner	0.62	(0.11–1.13)	0.02
Mother has been in trouble with the law	−0.24	(−1.51–1.03)	0.71
Mother has been hospitalised	0.14	(−0.08–0.36)	0.23
Mother has taken cannabis	−0.49	(−1.00–0.02)	0.06
Mother is in paid employment	−0.3	(−0.50–−0.10)	0.00
Mother drinks at least once per day	−0.27	(−0.58–0.04)	0.09
Financial difficulty score	0.19	(0.17–0.21)	0.00
Partner has been emotionally cruel to mother or child	0.69	(0.36–1.02)	0.00

**Table 4:** SDQ scores at 81 months (OLS).

	Estimate	Std. Error	*p*-value
(Intercept)	6.44	(5.93–6.95)	0.00
Mother has contact with social workers	2.24	(1.67–2.81)	0.00
Child is male	0.93	(0.71–1.15)	0.00
Mother has been depressed	1.65	(1.38–1.92)	0.00
Mother's has been married	0.65	(−0.49–1.79)	0.26
Mother's has been divorced	−0.12	(−0.92–0.68)	0.76
Mother has a partner	−0.44	(−0.95–0.07)	0.09
Mother has been in trouble with the law	1.57	(−0.25–3.39)	0.09
Mother has been hospitalised	0.3	(−0.03–0.63)	0.07
Mother has taken cannabis	0.13	(−0.44–0.70)	0.65
Mother has gotten a new job	0	(−0.27–0.27)	0.99
Mother has lost a job	1.62	(0.97–2.27)	0.00
Mother drinks at least once per day	0.77	(−0.39–1.93)	0.19
Mother has been in major financial difficulty	0.91	(0.52–1.30)	0.00
Partner has been emotionally cruel to mother or child	0.73	(0.28–1.18)	0.00
Mother was teenager at birth of study child	2.04	(1.20–2.88)	0.00

**Table 5:** Difference in SDQ scores between children with and without social worker contact at different quantiles.

		Lowest 25% SDQ			Highest 25% SDQ	
	Estimate	CI95%	*p*-value	Estimate	CI95%	*p*-value
42 months	1.13	(0.50–1.76)	<0.01	1.57	(0.53–2.60)	<0.01
81 months	1	(0.45–1.55)	<0.01	3	(0.79–5.21)	<0.01

Whilst differences in SDQ scores are higher for children whose mothers had contact these elevated scores are relatively modest considering the unconditional variance in SDQ scores in the ALSPAC population. For instance, children whose mothers had contact had average scores that were 0.35 standard deviations higher at 42 months and 0.48 standard deviations higher at 81 months after accounting for other predictors. In comparison, previous UK research has defined the clinically relevant cut-off for SDQ scores as the top 10% of children--which is roughly 1.28 standard deviation above the average (see Kelly et al., 2009, 131).

## 6 Discussion

To our knowledge, this study is the first to present evidence on the scale of false reporting of social worker contact in large scale studies. As such, our results may have implications for past and future social work research using large scale observational studies. Based on a small sub-sample of the ALSPAC population linked to the child protection register, we found substantial levels of false-negative reporting about contact with social workers. There are several possible explanations for this. First, errors could have been introduced through the linkage process (i.e. linkage error introduced false positive links to social work contact). Second, misattribution bias could be a factor, and we noted a larger probability of negative reporting when the participant was asked to recall over 18 rather than 12 months. Third, on this socially sensitive topic, mothers may under-report due to social desirability bias. These rates of reporting disparity were found amongst mothers in contact with social workers for the most serious reasons: child maltreatment and neglect are associated with the highest levels of stigma within society. It is therefore plausible (yet not measurable with the available information) that false reporting levels are lower in women in contact with social workers for other – less stigmatising - reasons.

Reporting rates may be influenced by local factors, which would mean our findings cannot be generalised more broadly to the UK. This could result from local social work policy being particularly adversarial, resulting in participants consciously failing to self-report social worker contact due to fear of further social worker involvement (although there is no evidence to suggest this is the case), or, that reporting is impacted by socioeconomic and demographic characteristics in ALSPAC, which has a population with lower levels of deprivation and ethnic diversity than the national population (Boyd et al., 2013). For this paper, we did not conduct any further analyses of the factors associated with underreporting due to the very small number of unit available for analysis.

Our analysis found that mothers who were hospitalised or started drinking alcohol at least once a day, in the previous 12 months predicts a greater likelihood of social work contact. Mothers who married for the first time in the previous 12 months were less likely than single mothers to have social work contact. Emotional and behavioural problems were markedly worse among children with maternal social work contact than among other children in the study, after controlling for observed adversities. Quantile regression suggests that differences exist for children across the range of SDQ scores. The results suggest that the disparity is not entirely caused by particularly vulnerable children, especially those suffering child maltreatment, driving up the mean SDQ scores of children with maternal social worker contact. In general, children whose mothers have social worker contact have significantly higher SDQ scores than others experiencing similar adversities.

As noted at the outset, it is important to be cautious when interpreting these findings, especially with regards to whether they indicate the causal effects on children of mothers’ contact with social workers. Beyond the broader challenges of capturing and measuring risk, vulnerability, adversity and outcomes, and leaving aside the issue of confounding factors, it may simply not be reasonable to expect a positive impact on a child’s emotional and behavioural state to result from social work contact with their mother for reasons which may or may not be associated with the child. The time-frames involved may also be too short for any benefits of social work intervention to accrue to children. It is further possible that the interaction of a set of multiple adversities commonly found in children whose families have social worker contact, combined with diverse assets, vulnerabilities and levels of resilience, creates a non-additive effect on children’s well-being (Davidson et al., 2012, Sabates and Dex, 2015). In addition, we had limited our predictors of SDQ to include the same—or equivalent—set of variable as used in our analysis of social work contact.

As noted in the methods section, the social work contact variable is lacking in specificity. We do not know the degree, nature or depth of involvement. The variable picks up characteristics of vulnerability not present in other variables but it is possible that these differences may not have been detected if a longer list of covariates had been used or if adversities not assessed in ALSPAC had been captured in the data.

Notwithstanding these limitations, the key strengths of the study are that it considers the wider population of families who have social work contact and issues of underreporting of these cases in surveys. These families and children have received relatively little research attention, compared for example with children placed in out-of-home care. This study’s findings are broadly similar to those from our analyses of the Millennium Cohort Study (MCS), the British Household Panel Study and the Longitudinal Study of Young People in England (Henderson et al., 2015, Henderson et al., 2016, Henderson et al., 2016, Zhang et al., 2016). Our work using MCS is the closest comparison, since the observed outcome is also SDQ score, albeit change in SDQ over time rather than SDQ scores at particular times. From the MCS, we found that martial status and homelessness was a were strong predictors of social work support—the latter was included in our ALSPAC analysis. We also found that after controlling for other factors that social work support had a negative association with SDQ (-0.61)—albeit our estimates had wide confidence intervals (CI95%: −1.45 to 0.23).

This study adds to our knowledge from MCS in three ways. First, it allows for comparison of the self-report measure with linked administrative data. Second, the ALSPAC self-report question, which asks about ‘contact’ with a social worker, is broader than the MCS question which asks about ‘seeking or receiving advice or help’ and so may miss social work contact imposed rather sought out. Third, since ALSPAC recruited families one decade earlier than MCS, the consistency of these effects over time and across different cohorts of children is indicative of the persistence of the observed differences.

Given the study’s limitations, especially about the unspecific measure and underreporting of social work contact, and in light of the ongoing importance of research on families receiving social work services, we believe that existing datasets on social work users need to be augmented by other data sources. This could take the form of new data collection, such as a dedicated cohort study of UK social work service users (akin to the US the National Survey of Child and Adolescent Well-being). Although, more survey data would not necessarily alleviate data quality issues. A more straightforward and cost-effective solution would be to allow existing cohort studies to conduct routine linkages to administrative social work data and to follow up this population of interest through targeted quantitative and qualitative data collection, using innovative outreach approaches if needed. Triangulation between these different data sets would also greatly enhance the explanatory power of any future investigations.

## 7 Conclusion

The results of this study indicate that social work contact is associated with greater emotional and behavioural problems in children. For reasons discussed, interpretation of these findings needs caution. One interpretation may be that being allocated a social worker is an indicator of acute and/or chronic problems that are not captured in the cohort study data. Another might be that the degree of difficulties faced by children whose families, for whatever reason, have social worker contact, is greater than the simply the sum of known adversities. Whichever way we understand it, it seems clear that other professionals working with children, such as teachers, need to be alert to the vulnerability of those whose families have social work contact. It may be that the continuing prioritisation of child protection in local authority practice (Featherstone, White, & Morris, 2014) comes at the cost of greater attention on children in need for other reasons, given that their outcomes do not appear to reflect improvement over time. More broadly, it seems clear that more systematic research is needed on this population of families, their needs and their outcomes. Finally, we believe that the self-reporting of social work contact in cohort studies poses a limitation that should be addressed through triangulating with linked records. A recent study linking ALSPAC with educational data and records on looked after children provides a considerable step towards this goal (Teyhan, Boyd, Wijedasa, & Macleod, 2019).

## CRediT authorship contribution statement

**Meng Le Zhang:** Conceptualization, Methodology, Formal analysis. **Andrew Boyd:** Data curation, Formal analysis. **Sin Yi Cheung:** Conceptualization, Methodology, Funding acquisition. **Elaine Sharland:** Conceptualization, Funding acquisition. **Jonathan Scourfield:** Conceptualization, Supervision, Funding acquisition.

## Declaration of Competing Interest

The authors declare that they have no known competing financial interests or personal relationships that could have appeared to influence the work reported in this paper.
